# Length of hospital stay prior to ICU admission and outcome

**DOI:** 10.1186/cc9885

**Published:** 2011-03-11

**Authors:** K Simpson, G Williams, T Quasim

**Affiliations:** 1 Glasgow Royal Infirmary, Glasgow, UK

## Introduction

We hypothesised that for the general ICU population, a longer length of hospital stay prior to ICU admission was associated with a poor outcome. Previous work in specific ICU populations has suggested that a longer length of hospital stay prior to ICU admission is associated with a higher mortality [1,2], and longer and therefore more costly ICU stays [3]. We undertook an evaluation of the relationship between pre-ICU length of hospital stay (LOS), and hospital mortality over a 1-year period.

## Methods

Using prospectively collected data, we undertook a retrospective evaluation of all patients admitted to the ICU of Glasgow Royal Infirmary from 1 August 2008 to 1 August 2009. Patients were identified from Wardwatcher (Critical Care Audit Ltd). Only the initial event was included in those patients with readmissions during the same hospital stay. The patients were divided into hospital survivors (Group A) and nonsurvivors (Group B). Statistical analysis was performed using SPSS version 15.0 for Windows (SPSS Inc, Chicago, IL, USA). Medians, interquartile ranges (IQRs) and Mann-Whitney U tests were applied as appropriate.

## Results

A total of 419 patients were admitted during the study period. After excluding those with missing data and the outliers, 397 were included in the data analysis. There were 268 in the survivor group (Group A), and 129 in the group that died (Group B). Median patient age: Group A, 50 (IQR 36 to 66), Group B, 62 (IQR 50 to 70), *P *< 0.001. Median APACHE II scores: Group A, 15 (IQR 10 to 20), Group B, 23 (IQR 18 to 29), *P *< 0.001. Median predicted hospital mortality (%): Group A, 15.9 (IQR 6.3 to 31.6), Group B, 46.8 (IQR 30.8 to 67.4), *P *< 0.001. Median pre-ICU LOS (days): Group A, 1 (IQR 0 to 2), Group B, 1 (IQR 0 to 4), *P *= 0.001. Median ICU LOS (days): Group A, 2 (IQR 1 to 6), Group B, 2 (IQR 1 to 7), *P *= 0.297. Median hospital LOS (days): Group A, 18 (IQR 7 to 36), Group B, 8 (IQR 3 to 23), *P *< 0.001.

## Conclusions

In our cohort, the critically ill patients who survived to hospital discharge were younger, were less severely unwell and had a significantly shorter length of stay prior to ICU admission. What cannot be determined from this study is the bias of individual clinicians when seeing referrals. Assuming we admit the patients we anticipate to have the best chance of hospital survival, patients with a longer length of hospital stay prior to ICU appear to have worse outcomes.
